# Detecting radioactive particles in complex environmental samples using real-time autoradiography

**DOI:** 10.1038/s41598-024-52876-w

**Published:** 2024-03-05

**Authors:** Joyce W. L. Ang, Arthur Bongrand, Samuel Duval, Jérôme Donnard, Ester M. Jolis, Satoshi Utsunomiya, Kenta Minomo, Risto Koivula, Marja Siitari-Kauppi, Gareth T. W. Law

**Affiliations:** 1 Department of Chemistry, Radiochemistry Unit, The University of Helsinki, 00014 Helsinki, Finland; 2 Singapore Nuclear Safety and Research Initiative, National University of Singapore, Singapore, 138602 Singapore; 3 AI4R, 2 Rue Alfred Kastler, 44307 Nantes, France; 4 IMT Atlantique, Nantes Université, CNRS, 44000 Nantes, SUBATECH France; 5 Circular Economy Solutions Research Laboratory, Geological Survey of Finland GTK, 02151 Espoo, Finland; 6 Department of Chemistry, Kyushu University, 744 Motooka, Nishi-Ku, Fukuoka, 819-0395 Japan

**Keywords:** Environmental monitoring, Nuclear chemistry, Natural hazards

## Abstract

Radioactive particles often contain very high radioactivity concentrations and are widespread. They pose a potential risk to human health and the environment. Their detection, quantification, and characterization are crucial if we are to understand their impact. Here, we present the use of a real-time autoradiography gaseous detector (using parallel ionization multiplier) to expedite and improve the accuracy of radioactive particle screening in complex environmental samples. First, standard particles were used to assess the detector capabilities (spatial resolution, spectrometry, and artefact contributions), then, we applied the technique to more complex and environmentally relevant samples. The real-time autoradiography technique provides data with a spatial resolution (≲100 µm) suitable for particle analysis in complex samples. Further, it can differentiate between particles predominantly emitting alpha and beta radiation. Here, the technique is applied to radioactive cesium-rich microparticles collected from the Fukushima Daiichi nuclear exclusion zone, showing their accurate detection, and demonstrating the viability of real-time autoradiography in environmental scenarios. Indeed, for more complex samples (radioactive particles in a less radioactive heterogeneous background mix of minerals), the technique permits relatively high selectivity for radioactive particle screening (up to 61.2% success rate) with low false positive percentages (~ 1%).

## Introduction

Radioactive particles are localized aggregates of radioactive atoms. Due to their discrete nature (micrometer to millimeter size), and often high specific radioactivity, these particles give rise to a heterogeneous distribution of radionuclides in environments compared to the background^1^. Radioactive particles can be released from various sources, including nuclear accidents, weapons testing (e.g., nuclear weapons, depleted-uranium munitions), or other industrial processes such as uranium mining and milling e.g.,^2–4^. As a result, they can exist in many different forms and with different properties (in terms of radionuclide content, radionuclide speciation, solubility etc.). Recently, there has been renewed interest in radioactive particle detection techniques. This reflects the realization that significant amounts of radioactive particles were released during the Fukushima Daiichi nuclear power plant (FDNPP) accident^5,6^, and current increased risks presented by war and terrorism^7,8^. There is a clear need to better detect and isolate radioactive particles from complex samples, preferably in less time to allow for effective emergency and forensic response, as well as risk mitigation and clean-up.

Release of radioactive particles into the built and natural environment has been widespread^5,9^. There was a significant release of uranium oxides, corium (materials formed from nuclear reactor core meltdown), and fission product condensate particles from the 1986 Chernobyl nuclear reactor explosion and fire^1,10^. More recently, the FDNPP accident has highlighted that radioactive particles are also likely a major output of lower energy nuclear accidents. The damaged FDNPP reactor units released significant amounts of glassy radioactive cesium-rich microparticles (CsMPs) into the environment^4,11^ due to loss of reactor containment and molten core concrete interaction (MCCI)^12^. Other significant release pathways for radioactive particle release to the environment include industry (e.g., mining and processing industries) and warfare. For example, there are concerns over uranium particle release from mines^13^, and there has been significant use of depleted uranium (DU) weapons in several warzones (Iraq and Kuwait, Bosnia-Herzegovina, and potentially Ukraine)^14,15^.

Radioactive particles present concerns for human and ecosystem health^16^. External radiation exposure to highly radioactive particles could cause skin lesions^17^. Internal exposures (from inhalation, ingestion, and contaminated wounds) could lead to further radiation-related illnesses. Particles (with size classification of < PM_10_) can be inhaled or ingested, and can be retained in the body^18–20^. Long-term exposure to radiation doses increases risk of chronic radiation-related illnesses (e.g., cancers and genetic mutations). Recently, CsMPs were found to induce inflammatory signaling and DNA damage responses in human primary lung fibroblast and bronchial epithelial cell lines^21^. Uranium-containing particles from mining have also been shown to induce pulmonary and vascular toxicity^13^. In the environment, radioactive particles can also control radionuclide transfer in the biosphere (e.g., Sr-90 cycling after the Chernobyl accident)^22–24^.

Existing techniques for the detection and subsequent isolation of radioactive particles can be tedious and time consuming. Often, samples (e.g., soils, air filters etc.) undergo sample splitting. Here, samples are separated into two parts of equal mass. The part with increased radioactivity is retained and further split. This process is repeated until a small sample containing highly radioactive particle(s) is achieved^25^. This technique usually employs the use of dry separation with imaging methods such as phosphor screen autoradiography^26^ and solid-state nuclear track detection (SSNTD)^1^, or wet separation with gamma spectroscopy^27^. Gamma spectroscopy is often used as a pre-screening technique to identify radionuclides contained in the particle and quantify their activities^28^. However, it does not provide spatial information on the particles needed for locating and extraction. Phosphor screen autoradiography provides high resolution two-dimensional images of radioactive emissions from a sample, while SSNTD allows for quantification and location of alpha-emitting particles^25,29^. However, these imaging techniques are slow (analysis takes days) and prone to error. Even after days of analysis, resulting data could still be useless due to under or over exposure, or when one finds that the sample does not contain radioactive particles. Under or over exposure situations are particularly common for environmental samples with unknown radioactivity, resulting in repeated, time-consuming measurements with differing exposure times. In addition, these techniques only provide rudimentary data: whilst they provide the locations of the radioactive particles in a sample, information on the number of radioactive particles can be hard to evaluate due to high background radioactivity. Further, the above quantification techniques yield no isotopic information. To obtain isotopic information, the particles have to be subjected to further measurements with gamma spectroscopy (for gamma-emitting radionuclides)^30,31^ or mass spectrometry (which can be destructive or at best semi-destructive)^32–34^.

Recent development in real-time autoradiography in direct counting mode, using micro-pattern gas detectors (MPGDs), has shown potential for application towards detection of radioactive particles in environmental samples^35^. The real-time function eliminates the trial-and-error process inherent to phosphor screen autoradiography and time wasted on samples that do not contain radioactive particles. This permits faster screening and detection suitable for time-sensitive scenarios (e.g., after nuclear accidents, warfare, or illicit use of nuclear materials). In this study, we demonstrate use of a state-of-the-art MPGD with a parallel ionization multiplier (PIM) structure (BeaQuant^36,37^) to detect radioactive particles in complex samples. Previous studies have shown that the BeaQuant system is able to spatially distinguish between two individual beta-emitting radioactive particles in simplistic standard samples^35^. However, the BeaQuant can also detect both alpha and beta emissions, and perform spectrometry between alpha- and beta-emitters^38^, and within alpha-emitters^39^; it is thus possible to obtain autoradiographs of different radionuclides in the same acquisition, if the energy difference between them is substantial. This capability could provide additional information on radionuclides present in particles in complex samples.

Despite recent BeaQuant system development^35^, further work is required to establish this technique for use in radioactive particle monitoring, quantification, and extraction from complex environmental samples. Given the small sizes of radioactive particles, it is exceedingly difficult to find the particles accurately and precisely. Therefore, it is imperative to quantify the spatial resolution of the BeaQuant system to understand the detector limitations in measuring such particles. Further, since BeaQuant uses high voltages (a few kV), sample defects such as trapped dust or air pockets can introduce discharges into the detector, causing false signals (termed as artefacts). These artefact signals look like ‘hot’ spots on autoradiographs and could be confused with real data generated by radioactive particles. Assessments of the signals produced by artefacts vs. real particles must be made to differentiate them. Finally, our past study demonstrated that BeaQuant could detect radioactive particles reliably through measuring simple, standard samples (artificially synthesized particles in a non-radioactive background), however, this concept has not been extended to more complex environmental samples (e.g., when highly radioactive particles are present against a background of heterogeneous radiation).

Reflecting the above, the overarching objective of this study was to establish the BeaQuant system for detecting radioactive particles in complex environmental samples. To achieve this, we used both Monte Carlo simulation with a GEANT4 toolkit^40^ and collected autoradiographs of simplistic standard samples to quantify the spatial resolution of the BeaQuant system with regards to particle detection. We then created a library of data from analysis of different particle types (those that predominantly emitted alpha and beta radiation), more complex environmental samples that included background radioactivity, and artefacts, to ground truth this technique. Ultimately, we show that the BeaQuant system can be confidently used to detect and quantify radioactive particles in complex environmental samples.

## Results and discussion

Several types of radioactive particles were used in this study: (i) synthesized Cs labeled particles, (ii) low enriched uranium (LEU) oxide particles, and (iii) CsMPs released from the FDNPP accident (see Materials and Methods, Section "Preparation of radioactive particles" for more details on each type of particle). The synthesized Cs particles and LEU particles were used as models for radioactive particles that predominantly emit beta and alpha radiation, respectively. These ‘model’ particles were used to investigate the spatial resolution (by comparing the output to the simulation results) and energy spectra profiles (for beta and alpha emissions) as registered by the BeaQuant detector. Samples prepared for BeaQuant analysis can also contain defects that could result in artefacts. Careful characterization of the model samples and subsequent measurement on the BeaQuant allowed us to evaluate artefact contributions to BeaQuant data.

To further evaluate whether the BeaQuant can accurately identify radioactive particles in environmentally relevant samples, the synthesized Cs particles and LEU particles were also added to more complex samples that contained an elevated background due to the presence of other radiolabeled minerals. Finally, CsMPs were used to determine the application of the BeaQuant to real environmental materials collected from a nuclear accident exclusion zone.

### Spatial resolution studies with gaussian blurring

**Figure 1:**
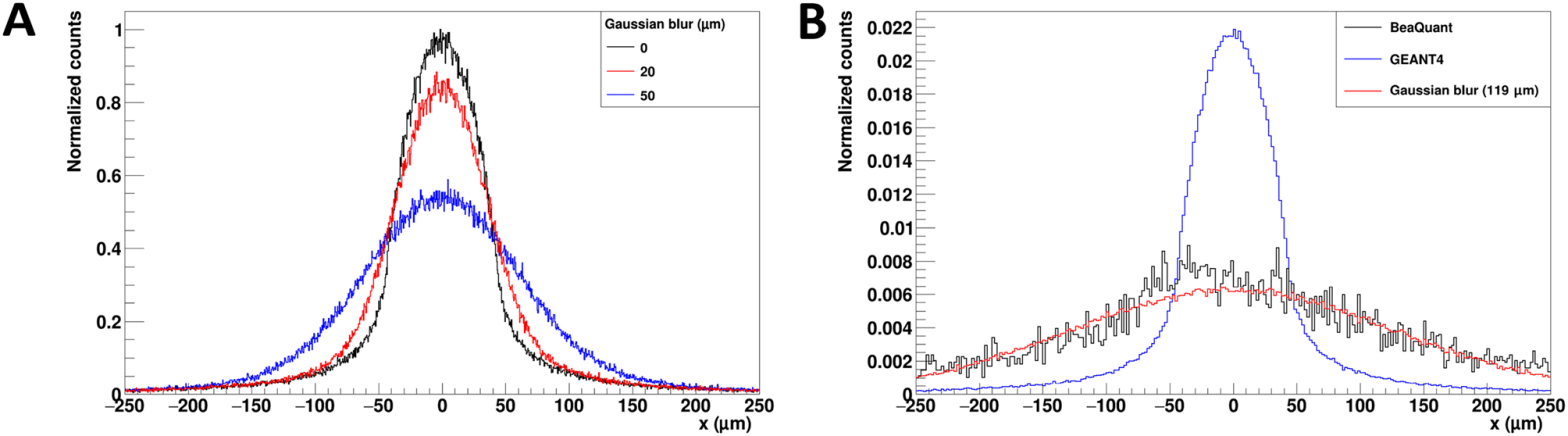
Gaussian filter applied to our simulation results. (**A**) Comparison between the Gaussian blurring, with standard deviations of 20 and 50 μm respectively, to the original simulation. The plots were normalized to the maximum peak of the original simulation. (**B**) An example of the Gaussian filter (standard deviation = 119 μm) used in the simulation to fit one of the BeaQuant data from a Cs-134 standard particle. The plots were normalized to the integral of each peak, respectively.

To determine the spatial resolution and peak broadening effect due to the detector, we applied Gaussian blurring filters (adding a Gaussian mathematical function to broaden the peak) to the simulation results and compared it to the experimental data acquisition for 5 Cs-134 and 5 LEU particles, respectively. Figure 1 shows an example of the Gaussian blurring for a Cs-134 particle.

All 10 Gaussian blurring filter standard deviations are listed in Table 1. The average Gaussian filter standard deviation for Cs-134 particles is 124 ± 15 μm, and 31 ± 12 μm for LEU particles. The standard deviation obtained for the LEU particles is substantially lower than the Cs-134 particles due to the difference between their radiation types; LEU is an alpha-emitter while Cs-134 is a beta-emitter. Since alpha particles (~ 4 atomic mass units) scatter less than beta particles (~ 0.000543 atomic mass unit) when traversing through the detector, we expect less substantial peak broadening effects (quantified by the standard deviation of the Gaussian filter) for LEU.

**Table 1:** Full-width at half maximum (FWHM) obtained from each BeaQuant data and simulated peak, and the standard deviation of the Gaussian blurring filter required to fit the simulation results into the BeaQuant experimental data. The uncertainty of the FWHMs were obtained from the fitting parameter error.

Particle type	BeaQuant FWHM (µm)	Simulated FWHM (µm)	Gaussian blurring (µm)
Cs-134	307 ± 5	79.2 ± 0.2	119
	290 ± 10	38.5 ± 0.1	116
	279 ± 8	69.5 ± 0.2	107
	350 ± 20	32.4 ± 0.1	141
	342 ± 9	34.1 ± 0.1	138
LEU	90 ± 8	24.8 ± 0.2	33
	87 ± 6	22.7 ± 0.1	32
	60 ± 10	35.8 ± 0.2	16
	135 ± 8	59.3 ± 0.3	47
	78 ± 9	33.0 ± 0.2	28

As shown in Table 1, each standard deviation is within the micrometer size ranges, which confirms that it is possible (in general) to detect radioactive particles with a good spatial resolution of ~ 100 μm for beta-emitting particles and < 100 μm for alpha-emitting particles. Moreover, the variation in standard deviation is consistent within the type of radioactive particle. This suggests that this information can be used to predict and obtain information on radioactive particle size by deconvoluting the Gaussian blurring effect on the BeaQuant acquisition data.

### Cs-134 and LEU particle energy spectra profiles

**Figure 2:**
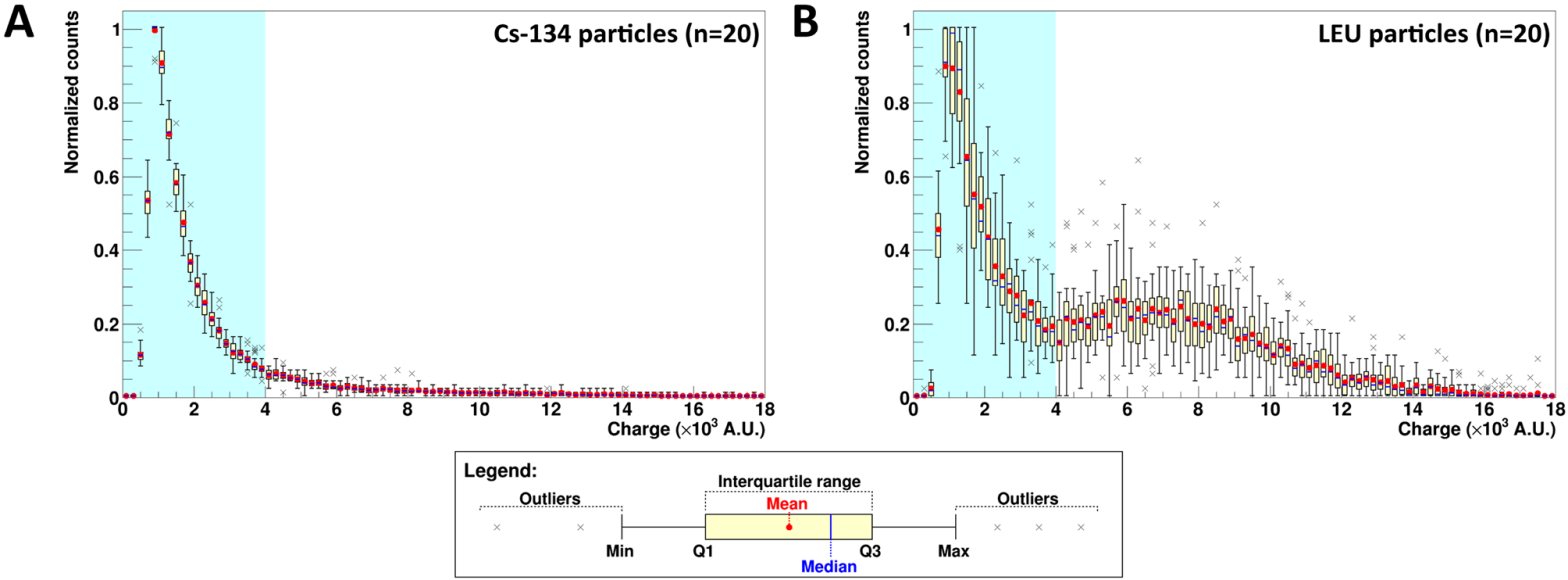
Electron energy deposited into the detector (represented by charge distribution acquired from the BeaQuant system) for emissions from (**A**) Cs-134 and (**B**) LEU particles, respectively. In each plot, 20 particles were used as datapoints for the candle plot. The light blue region highlights the lower energy range for the detection of beta particles, whereas the white region is typically the higher energy range for the detection of alpha particles. In the box-and-whisker diagram shown in the legend, the minimum is taken by subtracting 1.5 times of the interquartile range from the 25th percentile (Q1), whereas the maximum is taken by adding 1.5 times of the interquartile range to the 75th percentile (Q3).

We explored the possibility of conducting energy spectrometry between radio-Cs particles and LEU particles using the BeaQuant. To achieve this, the profile plots of normalized counts against charge signal, as measured by the detector, were collected from 20 Cs-134 particles and 20 LEU particles. Since the BeaQuant system functions like a proportional gas counter, the charge signal measured is directly proportional to the decay-induced energy deposition into the detector. All the data were compiled into candle plots presented in Fig. 2.

Comparison between Fig. 2A,B demonstrates the possibility to separate particles predominantly emitting beta vs. alpha radiation easily by spectrometry using the BeaQuant. The peak from the beta spectra measured by the detector exists in the lower energy ranges (light blue region with charge < 4 × 10^3^ A.U.), whilst the alpha peak can be found in the higher energy ranges (white region with charge ~ 4–16 × 10^3^ A.U.). This beta-like spectrum is in agreement with the spectra reported in past simulation and experimental findings for radio-Cs^35^. It is noted that Fig. 2B also contains a beta-like spectrum due to the beta-emitting daughter radionuclides in the U decay chain (e.g., thorium-234). Beta vs. alpha spectrometry via deconvolution is still possible for LEU particles found within the sample (< 19 µm, sample thickness limited to the 4.198 MeV alpha particle penetrating power emitted from U-238), since the alpha spectrum retains its Gaussian-like feature. However, due to energy loss of the alpha particles within the sample, the energy deposited into the detector and count rate are lower (peak shift to left and shorter peak).

### Artefact identification from Cs-134 and LEU particles

**Figure 3:**
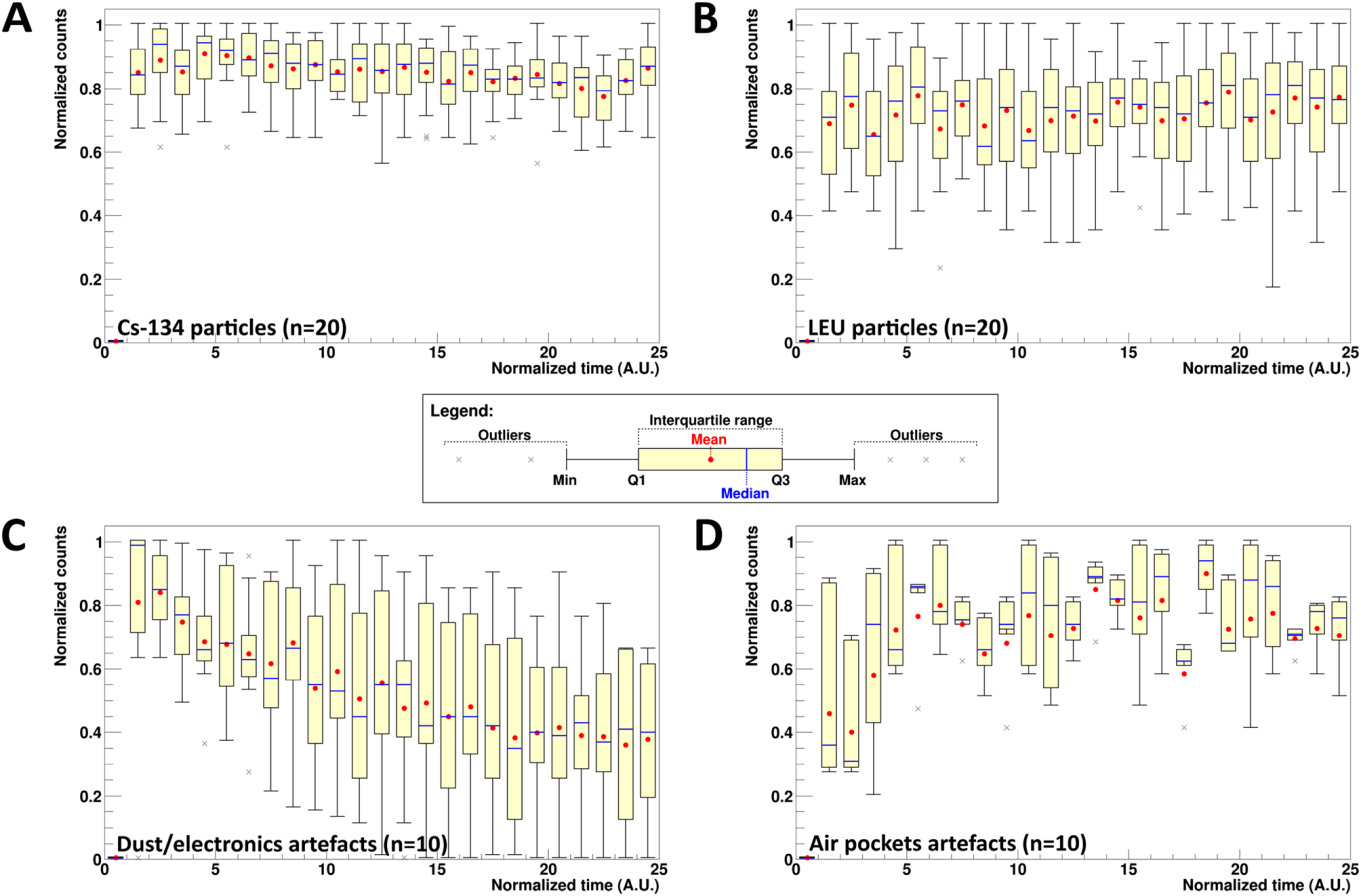
Time distribution of counts detected by the BeaQuant for (**A**) 20 Cs-134 particles (total acquisition time = 85,043 s), (**B**) 20 LEU particles (total acquisition time = 224,084 s), (**C**) 10 dust/electronics artefacts (total acquisition time = 85,043 s), and (**D**) 10 air pockets artefacts (total acquisition time = 84,948 and 237,557 s), respectively. The time was scaled by dividing the total acquisition time into 25 histogram bins. The reason for scaling the time to A.U. is due to the different acquisition times taken for the different datasets. The candle plot follows the same legend as Fig. 2.

Here, we present a novel method to distinguish artefacts (fake hotspots) from our actual Cs-134 and LEU particles. This method uses the difference between profile plots of normalized counts against the detector timestamp for particles vs. artefacts. In our study, a total of 20 Cs-134 particles, 20 LEU particles, 10 artefacts from dust/electronics, and 10 artefacts from air pockets in the sample were plotted in Fig. 3 to highlight differences.

From the mean (red datapoints) of the plots in Fig. 3, the time distribution is uniform and relatively constant for the Cs-134 and LEU particles. For the artefacts, the mean is erratic over time. The percentage differences between the minimum and maximum of the mean data for all 4 plots can be found in Table S1, Supporting Information. From the table, the mean data for particles fluctuate between ~ 16–18% whereas artefacts data vary largely (> 75%). This is because although radiation emissions are random, they follow a consistent decay trend given their half-lives. In this case, the half-lives of the Cs-134 and LEU particles are significantly longer than the BeaQuant acquisition time, therefore, they do not decrease over the measurement time. For particles with half-lives shorter or comparable to the acquisition time, an exponential decrease should be observed. This regular trend can be used for particle identification. Meanwhile for dust/electronics artefacts, these are usually due to an instant charge build-up, and as it discharges over time to reach stability, there is a reduction in the counts per unit time. As for artefacts caused by air pockets, we postulate that the charge builds up over time (~ 4 to 10 h, according to Fig. 3D), leading to an increase in counts per unit time until it reaches equilibrium. Another distinguishing factor in Fig. 3 is the spread of data in the *y-axis*, as shown in the interquartile range, minimum, and maximum, shown in the candle plots. The spread of data is lower for particles compared to artefacts. Energy spectra profiles of artefacts were studied to evaluate the possibility of identifying artefacts with spectrometry methods, however, we found that the differences were not sufficient to distinguish between artefacts and particles.

**Figure 4:**
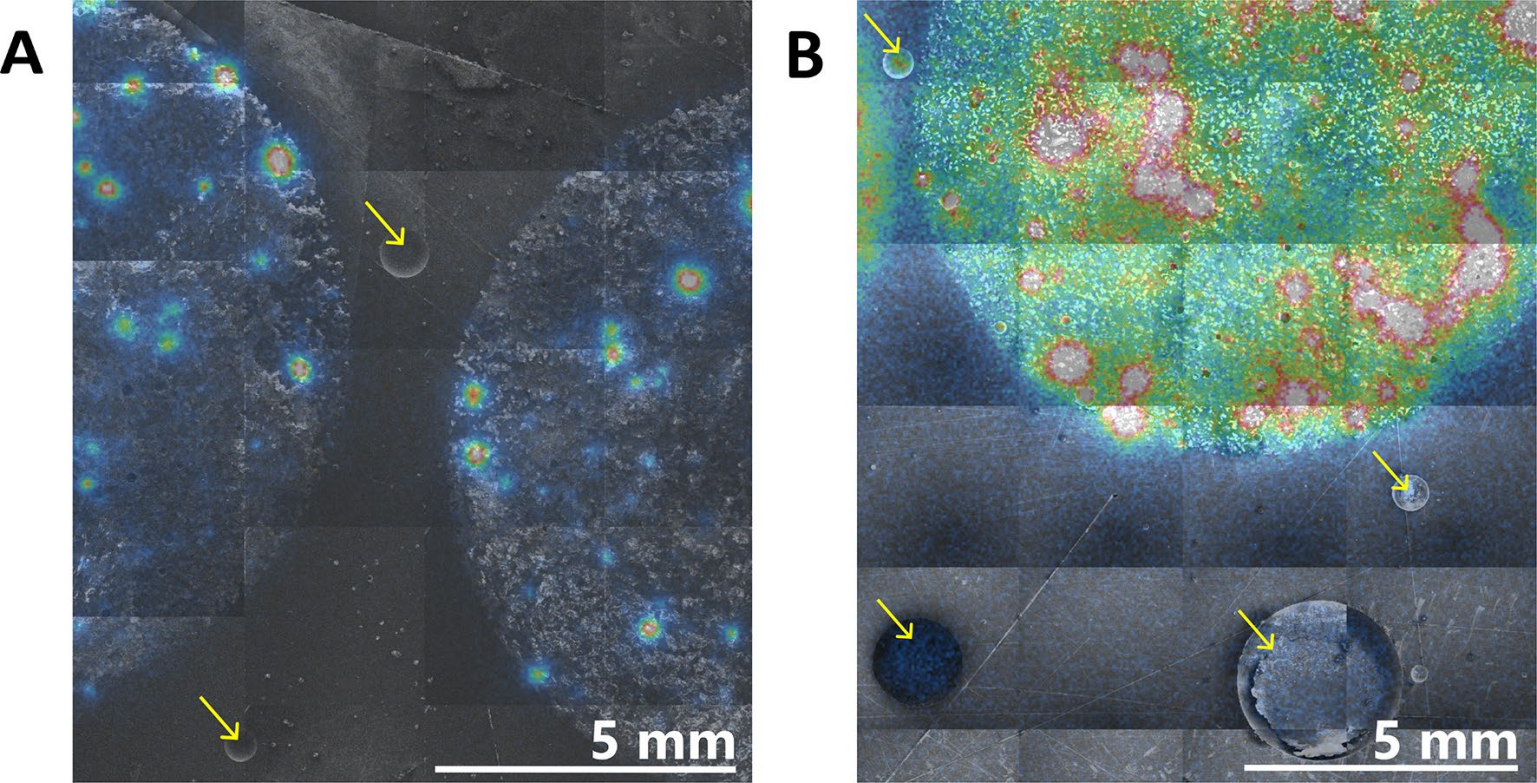
Comparison of artefact contribution from air pockets found in (**A**) a thin sample of thickness 70 ± 40 μm and (**B**) a thicker sample of thickness 680 ± 60 μm. Scanning electron microscopy (SEM) images were incorporated into montages and superposed onto corresponding autoradiographs to identify the position of the air pockets on the sample surface (yellow arrows) with respect to the autoradiograph signal. To ensure that the signal could only come from the air pockets, only air pockets well outside the range of the radioactive particles were selected for analysis.

We also observed that sample thickness affects the presence of artefact contribution from air pockets in the BeaQuant analysis (Fig. 4). Figure 4A shows two air pockets (as denoted by the yellow arrows) in a thin (70 ± 40 μm) sample that had no signal, whereas in Fig. 4B, four air pockets found in a thicker (680 ± 60 μm) sample contain slight signals. Generally, for samples less than 100 μm, there are almost no artefacts due to air pockets, however, for samples thicker than 100 μm, the air pockets are significantly deep enough to allow charge build-up, leading to the presence of artefacts.

### Assessing BeaQuant accuracy when measuring mineralogically complex samples

**Figure 5:**
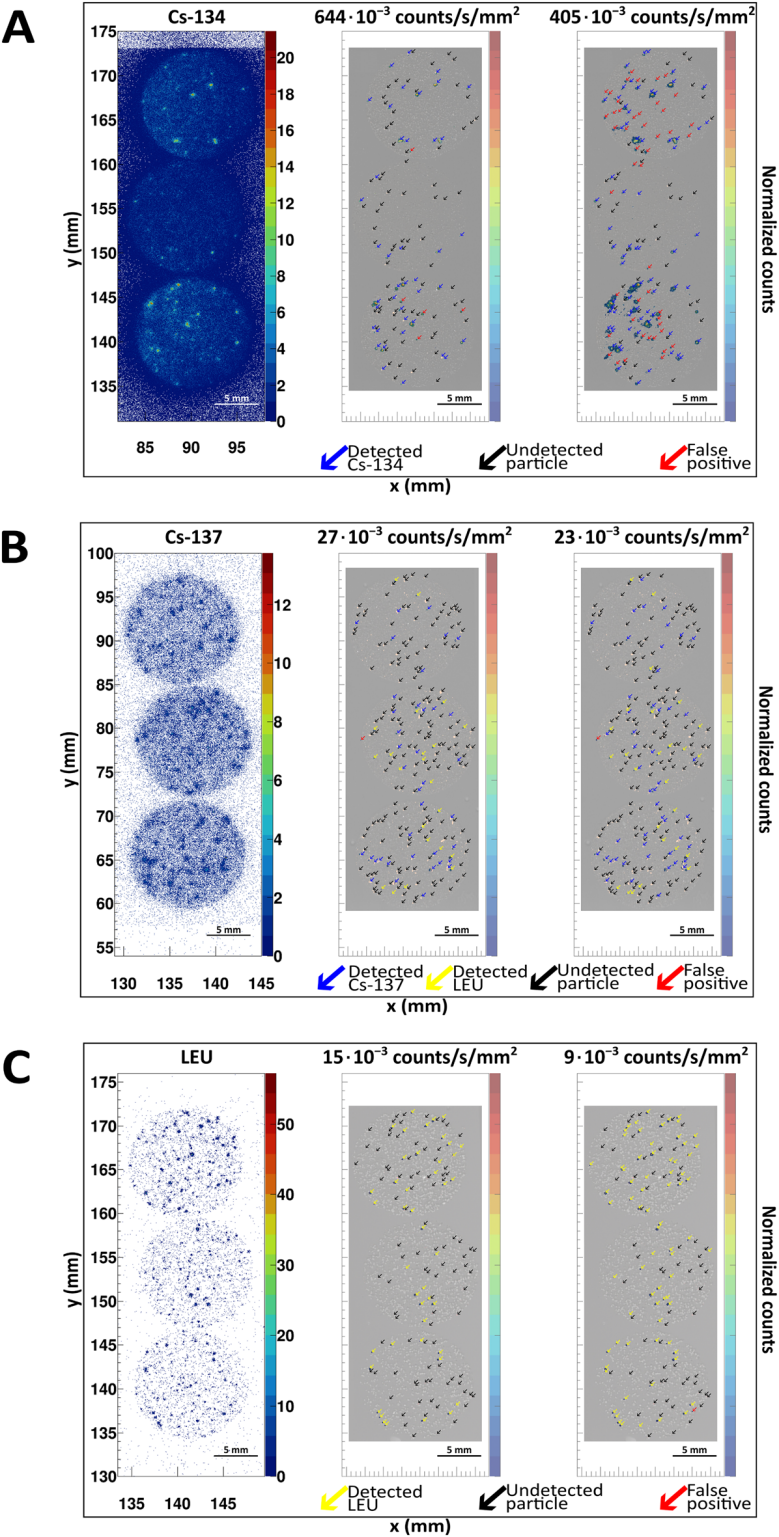
Comparison between the autoradiograph before and after imposing a surface activity concentration threshold (in counts/s/mm^2^) for the (**A**) Cs-134, (**B**) Cs-137, and (**C**) LEU complex samples. In each subfigure, the leftmost is the original autoradiograph obtained from the BeaQuant, while the other two images are the post-treated autoradiograph, with an implemented threshold cut algorithm (with the counts per second per mm^2^ values shown above the images) using the CERN ROOT environment. The higher threshold value is obtained by analyzing the average activity concentration of 20 particles, while the lower threshold is obtained by subtracting the average by the standard deviation. The post-treated images were overlaid onto the µXRF element map of Cu and U to identify the Cs and LEU particles, respectively. The non-superposed Cu and U maps for all three samples can be found in Figure S1, Supporting Information. For easy identification, arrows were used to pinpoint the position of particles or false positives. Blue arrows indicate the successful detection of radio-Cs with the algorithm (agreement between the BeaQuant and µXRF results) and yellow arrows indicate the successful detection of LEU with the algorithm. Black arrows indicate regions where the algorithm was unable to retain the BeaQuant signal when the µXRF element map shows the presence of a particle, and red arrows indicate false positives, which are regions where there is BeaQuant signal even though there are no presence of a particle shown in the µXRF element map.

Elemental maps from micro X-ray fluorescence (µXRF) analysis were used to locate the position of radioactive particles in the sample and compared to the autoradiography results to assess the accuracy of the BeaQuant in identifying radioactive particles (higher activity concentration) against an elevated background of other radiolabeled minerals (lower activity concentration). Figure 5 shows the autoradiograph and particle locations for three different samples: Cs-134, Cs-137, and LEU. We used Cs-134 as a high activity beta-emitter (0.5–55 MBq/g), Cs-137 as a lower activity beta-emitter (0.015–1.5 MBq/g), and uranium as an alpha-emitter (0.015–27 kBq/g). An energy threshold cut of 4 × 10^3^ A.U. (determined in Section "Cs-134 and LEU particle energy spectra profiles") can be imposed to separate Cs from LEU particles with spectrometry.

The Fig. 5 demonstrates that the threshold successfully cut out background noise, producing clean autoradiograph signals with distinct hotspots, regardless of the background radiation contribution from the other radiolabeled minerals. To evaluate the effectiveness of the algorithm, from the particles identified in Fig. 5, we tabulated the percentages of the algorithm’s successful detection (i.e., selectivity) out of all particles detected by µXRF analysis, and the percentages of false positive results (out of all signals retained in the algorithm). These percentages are reported in Table 2.

**Table 2:** Percentage of selectivity and false positives after treatment of the BeaQuant autoradiograph (Fig. 5). More details of each sample can be found in the Materials and Methods section, Table 3. The percentages were rounded up to 1 decimal place.

Samples	Algorithm threshold (·10^−3^ counts/s/mm^2^)	Selectivity (%)	False positives (%)
Cs-134 particles in a mixed background	644	35.3	10.9
	405	61.2	39.8
Cs-137 particles in a mixed background	27	25.8	1.6
	23	32.2	1.3
LEU particles in a U background	15	31.9	0
	9	52.9	1.4

These algorithm threshold values are almost double or 1 order of magnitude higher than the average counts/s/mm^2^ for a sample section reported in Table S2, Supporting Information. This observation is expected since the activity concentration between particles is ≥ 1 order of magnitude higher than the elevated background. From Table 2, the percentage selectivity ranges from 25.8–61.2%, which is highly dependent on the threshold implemented (inverse relationship). Since the threshold in this study is determined by analyzing 20 particles per sample, it is important to note that the selectivity might improve by increasing the sample population size of particles analyzed for activity concentration threshold. For the first two samples (Cs-134 and Cs-137), the radioactive Cs particles were identified in the µXRF maps using Cu, since the standard Cs particles were created by sorbing Cs onto copper hexacyanoferrate (Cu-HCF) particles. Here, we assume that all the Cu-HCF particles contain evenly distributed radioactive Cs. There might be cases where some Cu-HCF particles contain radioactive Cs with a lower activity concentration than the threshold, potentially leading to a lower selectivity, as evident in the Cs-137 sample.

Except for the Cs-134 sample, the false positive percentages are relatively low: ranging from approximately 0–1.6%, as shown in Table 2. This suggests that the BeaQuant is accurate with minimal overestimation when quantifying radioactive particles. One possible reason that the Cs-134 samples have larger false positive percentages is that the sorption of Cs-134 on the minerals contributing to the background is heterogeneous, such that some minerals grains (e.g., weathered biotite or illite) can have higher amounts of sorbed Cs. This observation is backed by Figure S2 in Supporting Information, which shows that in some regions with false positive results, there are presence of particles containing aluminum (found in the weathered biotite and illite but not in Cu-HCF) and iron (without Cu) in the µXRF element map. This emphasizes the challenges faced when studying particles in mineralogically complex samples. In these cases, the choice of threshold is important as there is a trade-off between improving the selectivity of the particles and producing more false positive results, as evident by the increase in false positive percentage from 10.9 to 39.8% (Table 2).

Another reason for the false positive signals in the Cs-134 sample might be due to the contribution by LEU particles in the complex samples, which fall below the detection limits of the µXRF U element map. In the Cs-137 sample, particle signals in the autoradiograph that do not correspond to the Cu element map often match with U. However, we note that the µXRF U map for the Cs-134 sample is noisier than the Cs-137 sample (Figure S1), and we are unable to locate any U particles in it. µXRF detection limits are highly dependent on the sample types and can vary due to factors such as bulk chemistry, material characteristics, and the influence of overlapping, interfering, and artefact peaks. It is also important to note that the Cs-134 sample contains ~ 10^3^ times more Cs radioactivity than the Cs-137 sample. As such, the radiation emitted from the Cs-134 samples may impact the µXRF detection limits.

Theoretically, the µXRF detection limit of U is typically less than 1–4%wt (1–4·10^4^ ppm). In contrast, the minimum detectable activity (MDA) of the BeaQuant for the U sample is 0.768 mBq/mm^2^, calculated following the method from Ang et al*.*^35^. This translates to approximately 200 ppm. Typically, radiometric detection methods such as autoradiography have a lower detection limit than other techniques. Consequently, the BeaQuant could pick up faint signals (Fig. 5C) from the background uranium sand (~ 600 ppm) that were not detected by the µXRF (Figure S1). The minimum detectable activity for Cs-134 and Cs-137 is 0.116 and 0.042 Bq/mm^2^, respectively, which is about 3 to 4 orders of magnitude below the surface activity of the radio-Cs particles measured in these complex samples. Although µXRF has a higher detection limit than the BeaQuant, this technique allows for accurate identification of the elements in the particle. As a result, the µXRF results complement the autoradiographs measured by the BeaQuant, and the techniques could be used in conjunction when establishing BeaQuant analysis of a new sample type.

### CsMP detection

**Figure 6:**
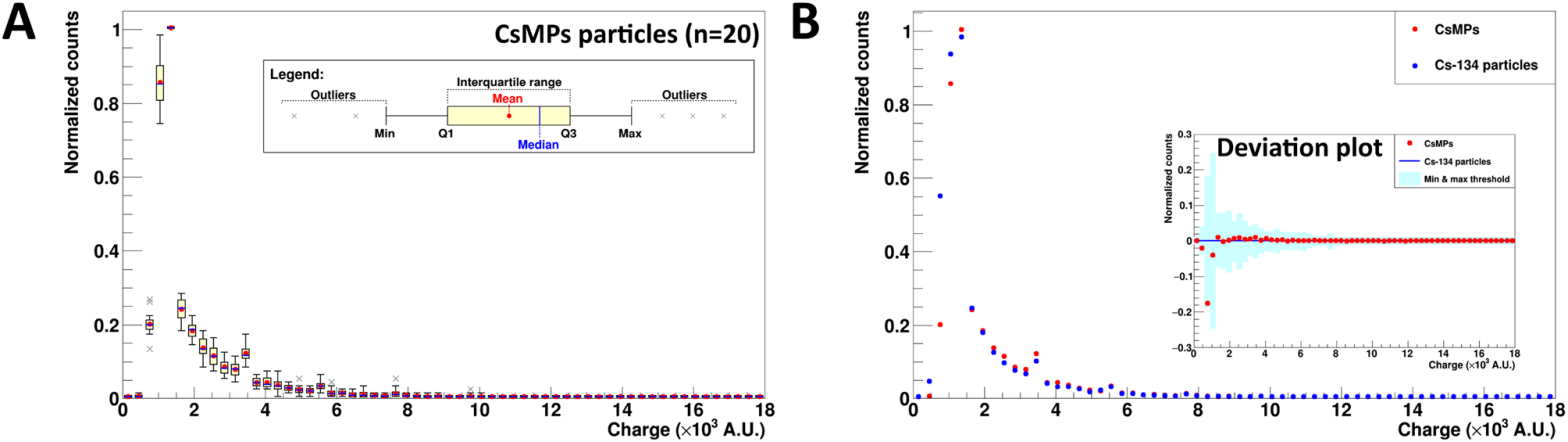
(**A**) Electron energy deposited into the detector from emissions of 20 CsMPs and (**B**) the comparison of the mean with the mean profile from 20 Cs-134 standard particles. The candle plot in (**A**) follows the same legend as Fig. 2. The deviation plot (of CsMPs from the Cs-134 particles) can be found in the inset of (**B**), where the light blue region represents the minimum (Q1 − 1.5 × interquartile range) and maximum (Q3 + 1.5 × interquartile range) threshold of each datapoint for the Cs-134 standard particle.

To ensure that BeaQuant is viable for real environmental applications (e.g., nuclear accident response), we measured 20 CsMPs isolated from soils collected in the Fukushima Daiichi nuclear exclusion zone. BeaQuant was successful in detecting the particles. The charge profile of all 20 particles is shown in a candle plot and compared against the Cs-134 standard particles (Fig. 6). From Fig. 6B, we note that there is minimal deviation (within the minimum and maximum thresholds in the light blue region) between both the CsMP and Cs-134 particles. This indicates that the radio-Cs found in the CsMPs was accurately detected, and within the expected energy spectra for Cs. As a result, BeaQuant can be used for the detection of environmentally relevant radioactive particles.

### Detector advantages, limitations, and future work

Autoradiography is widely used in radioactive particle research, to complement other techniques (e.g., gamma spectroscopy). Due to its high-resolution 2-dimensional imaging, it permits identification of particles from an elevated radioactive background. In contrast to phosphor screen autoradiography, the BeaQuant detector improves the time taken for obtaining these data. Equipped with real-time function, within the first few hours of acquisition, BeaQuant users can determine the usability of data and the optimal acquisition time. Acquisition time can also be adjusted to cater to different data requirements; a shorter time for obtaining particle positions, and a longer time for sufficient statistics in low radioactivity, spectrometry work, or noisy backgrounds. Development from this study highlighted (with the relevant example of CsMPs released from the FDNPP, n = 20 particles) the potential of BeaQuant in applications towards faster screening of radioactive particles. However, sample preparation can be improved to allow for better and faster extractions. Current methods in this study include sawing and polishing, which is time-consuming and may physically alter particles that are subjected to post-analyses. Some considerations would be to study the possibility of measuring particles from soil samples that are spread thinly on a plate and wrapped with a thin mylar film (like phosphor screen autoradiography) and to optimize particle measurements from aerosol filter samples. It is important to keep the samples as thin as possible (ideally ~ 10 µm) for the detection of alpha-emitting particles, because the penetration power of alpha particles is low. The spectrometry work here is only limited to Cs-134 and LEU particles. This could be extended to obtain a library of spectra for other common radionuclides (e.g., Sr-90, Pu isotopes) through use of suitable calibration standards. When scaling up the experiment to an actual scenario, the BeaQuant can take up to 6.9 g of soil per sample (with maximum field of view 23 × 23 cm^2^ and sample thickness 100 µm). Density-based clusterization algorithms such as Density-Based Spatial Clustering of Applications with Noise (DBSCAN) or Ordering Points to Identify the Clustering Structure (OPTICS) could be explored to process large datasets, to enhance the performance of quantification (speed of processing and increased sample population sizes for better threshold selection).

## Conclusions

With this study, we present a new and more accurate way of identifying and locating radioactive particles, using a state-of-the-art radiometric technique: real-time autoradiography with a gaseous detector. The BeaQuant has a good spatial resolution of 124 ± 15 μm and 31 ± 12 μm for Cs-134 particles and LEU particles, respectively. Moreover, it can perform spectrometry to identify particles that predominantly emits alpha or beta radiation, or both. This spectrometric ability was applied to CsMPs released from the FDNPP accident; the spectra recorded by 20 CsMPs matches the results from 20 Cs-134 particle standards. Although the BeaQuant is sensitive to artefacts, we show that it is possible to distinguish between artefacts and real radioactive particles. Hence, artefact contribution is not a significant problem for radioactive particle quantification with this equipment. For more mineralogically complex samples, the use of µXRF for elemental maps proved useful in extracting important information for better understanding the sample, in identifying the particles, and estimating the effectiveness of our algorithm for quantifying particle concentrations in an unknown environmental sample. This highlights that prior to measuring new environmental sample types with the BeaQuant, method development with corroborating analyses may be useful.

## Materials and methods

### Preparation of radioactive particles

To produce synthetic “standard” radio-Cs (Cs-134 and Cs-137) labeled particles, Cs-134 or Cs-137 were sorbed onto copper hexacyanoferrate (Cu-HCF) microparticles. The Cu-HCF particles were synthesized using the same method as in our previous work^35^. Briefly, K_4_Fe(CN)_6_ was added to CuNO_3_ under vigorous stirring. Cu-HCF slurry obtained from this reaction was washed with Milli-Q water, dried in an oven (at 70 °C) overnight, ground with a mortar and pestle, and sieved into a size fraction of < 25 µm. The Cu-HCF was split into 2 batches for Cs-134 and Cs-137 sorption respectively. The sorption of Cs-134 (Supporting Information, Figure S3) permitted creation of Cu-HCF particles that matched the activity per CsMP found in environmental samples (> 0.06 Bq per particle for particle sizes < 114 µm)^26^. A lower activity of Cs-137 was sorbed onto Cu-HCF to minimize creation of long-lived radioactive waste (Cs-137 half-life = 30.08 years).

For uranium particles, we used LEU (2.5% U-235) uranium oxide powder from the University of Helsinki inventory. The UO_2_ powder sample was obtained from the U.S. Department of Energy and contains U decay products such as Th-234.

Real environmental CsMPs were isolated from surface soils collected in July 2019 from the Yamada district, Futaba Town, Fukushima. Approximately 5 g of soil sample (sufficient to extract > 20 CsMPs for our study) was suspended in water, and droplets of the suspension were dispersed on an aluminum plate. The aluminum plate was then gently heated on a heating stage to dry it; phosphor screen autoradiography was then performed using an imaging plate, with a thin plastic sheet placed between the plate and screen to avoid contamination. Detected radioactive particles were extracted from the aluminum plate using a pipette, after each area showing the likely presence of a CsMP was wetted with water. More than 30 hot particles were collected and resuspended using this approach. Since the suspension also contained numerous clay particles that had lower Cs activities (typically ranging as high as ~ 10^6^ Bq/g as background)^26^, the dispersion and autoradiography described above were repeated 5 times to obtain a suspension without visible clay particles. The extracted CsMPs were then mounted onto scanning electron microscopy (SEM) sample holders using carbon tape. They were subsequently carbon coated to 20 nm for conductivity, which is important for BeaQuant analysis. The particles were measured on the BeaQuant to test and confirm that the detector was viable for measuring and locating radioactive particles from real environmental samples.

### Preparation of radiolabeled background

Radio-Cs was sorbed onto three different minerals (sieved to size fraction 50–100 µm): weathered biotite, illite (illite–smectite mixed layer in a 70:30 ratio from Slovakia ISCz-1, the Clay Minerals Society), and quartz to form a complex radioactive background matrix (similar to a mineral soil/sediment). The adsorption plots for creation of this material are presented in the Supporting Information (Figure S3). Among these three minerals, weathered biotite has the highest absorption capacity for Cs^41^. As a result, the mixture of minerals produced a heterogeneous distribution of radio-Cs in the background. The inclusion of weathered biotite incorporates some challenges in identifying radio-Cs labeled Cu-HCF particles, similar to ongoing challenges faced in environmental samples when differentiating CsMPs from Cs-clay.

To produce weathered biotite, 4 g of biotite (from Luumäki, Finland) was weathered with 1 L of 1 M NaCl (pH adjusted to 4 using 0.1 M HCl), at 40 °C for 20 days, following Kim et al.^42^. Afterwards, the weathered biotite was washed with Milli-Q water and dried at room temperature.

Uranium sand was produced by sorbing U(VI) (from a UO_2_Cl_2_ stock) onto ferrihydrite-coated quartz (Supporting Information, Figure S4)^43^. To produce ferrihydrite, we used the method of Hubbard et al.^44^. Here, 0.062 M ferric chloride was rapidly titrated with 0.4 M NaOH to a pH of 7.5. The resulting ferrihydrite suspension was aged overnight, and the precipitate was washed with Milli-Q water to remove residual salts. Acid-washed quartz was mixed with the ferrihydrite suspension overnight on a shaker. Afterwards, the remaining mixture was dried at room temperature under convection for 4 days with repeated stirring to ensure uniform coating of the quartz.

### Sample Preparation with Particles and Sediments

Particle and sediment samples were fixed with resin embedding. The epoxy resin used in this work is Araldite® M (1.038 g/cm^3^ at 25 °C) with hardener REN™ HY956 (1.02 g/cm^3^ at 25 °C), with a mass ratio of 5:1. The resin was subject to overnight curing before being sawn into sections of 1 mm thickness. These 1 mm sections were attached onto glass slides using the same resin and hardener mix before being polished with diamond plates of grit size 80, 500, 1200, and 2000 (MD-Piano, Struers). The final sample thickness (post-polishing) was quantified with a polarizing microscope or a micrometer dial indicator. Each thin section sample contains 3 circular resin embedded slices. Table 3 lists the different types of resin embedded samples prepared for this study, sample thicknesses, and the purpose for each sample.

**Table 3:** Summary of different resin embedded samples prepared. Weathered biotite is represented as WB here.

Sample	Components		Sample thickness (µm)	Purpose
	Particle	Background		
Cs-134 particles in non-radioactive quartz	Cs-134 Cu-HCF	Quartz	70 ± 40 680 ± 60 1000 ± 200	Spatial resolution Artefact identification
LEU particles in non-radioactive quartz	LEU UO_2_	Quartz	34 ± 6 40 ± 10 30 ± 8	Spatial resolution
Cs-134 particles in a mixed background	Cs-134 Cu-HCF LEU UO_2_	Cs-134 WB Cs-134 illite Cs-134 quartz	44 ± 4 84 ± 34 38 ± 14	Test ability to detect particles Differentiating alpha- from beta-emitters
Cs-137 particles in a mixed background	Cs-137 Cu-HCF LEU UO_2_	Cs-137 WB Cs-137 illite Cs-137 quartz	94 ± 11 73 ± 12 75 ± 6	Test ability to detect particles Differentiating alpha- from beta-emitters
LEU particles in a U background	LEU UO_2_	U quartz	58 ± 14 89 ± 17 90 ± 30	Test ability to detect particles

### Acquisition with the BeaQuant system

Real-time autoradiographs were acquired using a BeaQuant detector. The detector can measure alpha particles, beta particles, and other electrons (Auger/conversion electrons). It is insensitive to gamma radiation (~ 0.0025% efficiency at 185.7 keV) at room temperature and atmospheric pressure due to the low density and thickness of the gas media (90% Ne and 10% CO_2_). The working principles and schematic cross-section of a BeaQuant detector are detailed in Ang et al. (2023)^35^. Samples were first cleaned with compressed air to remove dust or impurities prior to loading them into the detector. For good counting statistics, the acquisition time for samples used in this study varied between 18 to 71 h. For all acquisitions in this study, the dead time contribution from the detector was insignificant. The acquisition data was analyzed with the software Beamage (version 3.4) and CERN ROOT (version 6.19/02)^45^.

In addition to reconstruction of the autoradiography, we investigated parameters such as the charge distribution collected by the detector, as well as the timestamps when the counts were detected. These parameters were used to identify the difference between artefact contribution and emissions from real radioactive particles. This information was assessed with CERN ROOT.

### Scanning electron microscopy (SEM)

A Hitachi S-4800 FESEM was used to obtain images of the sample surface (voltage: 5 kV; emission current: 5 µA) to identify air pockets in the sample surfaces caused by polishing or air bubbles from the cured resin. The SEM images were superposed to autoradiographs from the BeaQuant system using Inkscape imaging software. From this superposition of images, air pockets can be identified relative to the particles, such that it was possible analyze the parameters from the BeaQuant acquisition in that region.

### Micro X-ray fluorescence (µXRF)

A M4 Tornado (Bruker) µXRF was used to map the elemental composition of complex mixed mineral samples. The equipment has a 30 W rhodium (Rh) anode X-ray tube and two 30 mm^2^ silicon drift detectors with an energy resolution of < 145 eV at 275 kcps (measured on Mn Kα). The Rh X-ray source was operated under maximum energy settings (50 kV, 600 µA). The beam was focused by a polycapillary lens on a fixed spot size of 20 µm under 2 mbar vacuum. µXRF maps were obtained with a step size of 15 µm and a pixel dwell time of 15 ms. The resulting qualitative elemental maps (generated using the Bruker M4 software) allowed for identification and understanding of the spatial distribution of different particles. First, 20 particles were identified from each sample using the µXRF data, and the activity concentration (in terms of counts per second per mm^2^) for each of these particles was then recorded. Thereafter, a threshold cut computational code was developed with CERN ROOT to obtain less noisy autoradiographs which should pinpoint the exact location of any particles that fulfilled the activity concentration recorded. The computational code performs a raster scan of the entire autoradiograph according to a unit area of 5 × 5 pixels and tabulates the total counts in each unit area. Any area that contains counts lower than the desired threshold (activity concentration shown in Fig. 5) is discarded, retaining only regions with activity concentrations higher than the threshold. The autoradiographs from the BeaQuant system were superposed onto the µXRF maps using Inkscape for easy comparison, which was used to test if the BeaQuant system could pick out the locations from only the radioactive particles, even with a background radioactive contribution coming from mock sediments.

### Monte carlo simulations

In this study, an ad hoc GEANT4 (GEometry ANd Tracking 4, version 4.10.07) simulation was developed in order to quantify the spatial resolution of the detector. GEANT4 is a Monte Carlo C++ toolkit for simulating the transport and interactions of particles in matter, comprehensively modelling physical processes over a wide range of energies^40^. Previous studies with GEANT4 simulation results assumed an ideal detector and did not account for peak broadening effects (spread of data due to the electron travelling from the sample to the detector reading floor)^35^. The peak broadening effect is difficult to simulate directly because it requires the use of complicated computations accounting for the electric field within the detector. However, in this study, a Gaussian blurring effect was included in the simulated results to quantify the amount of peak broadening (spatial resolution) shown in the detector for Cs-134 and LEU particles. The Gaussian blurring filter was applied by adding a Gaussian function (of a certain standard deviation) using CERN ROOT to the spatial distribution outputs from the simulation. The standard deviation value was computed such that the peak FWHM from the simulated results matched that of the BeaQuant acquisition. This procedure was carried out for 5 Cs-134 particles and 5 LEU particles in total.

### Supplementary Information

Supplementary Information.

## Data Availability

The data that support the findings of this study are available within this article and its Supporting Information. Additional data are available from the corresponding author upon request.
