# Supplementary material for: Detecting radioactive particles in complex environmental samples using real-time autoradiography
Source: Sci Rep. 2024 Mar 5;14:5413. doi: 10.1038/s41598-024-52876-w (PMC10915129; doi:10.1038/s41598-024-52876-w)
Supplement: Supplementary file 1 — Supplementary Information. [file 41598_2024_52876_MOESM1_ESM.docx]

Supporting Information for:

**Detecting Radioactive Particles in Complex Environmental Samples using Real-time Autoradiography**

Joyce W. L. Ang^a,b,^*, Arthur Bongrand^c,d^, Samuel Duval^c^, Jérôme Donnard^c^, Ester M. Jolis^e^, Satoshi Utsunomiya^f^, Kenta Minomo^f^, Risto Koivula^a^, Marja Siitari-Kauppi^a^, Gareth T. W. Law^a,^*

^a^Radiochemistry Unit, Department of Chemistry, The University of Helsinki, Helsinki 00014, Finland

^b^Singapore Nuclear Safety and Research Initiative, National University of Singapore, 138602, Singapore

^c^AI4R, 2 rue Alfred Kastler, 44307, Nantes, France

^d^IMT Atlantique, Nantes Université, CNRS, SUBATECH, F-44000 Nantes, France

^e^Circular Economy Solutions Research Laboratory, Geological Survey of Finland GTK, Espoo 02151, Finland

^f^Department of Chemistry, Kyushu University, 744 Motooka, Nishi-ku, Fukuoka 819-0395, Japan

*Email: joyce.ang@helsinki.fi, gareth.law@helsinki.fi

**This PDF file includes:**

Figs. S1 to S4

Tables S1 to S2

**Table of Contents**

[**Figure S1: Copper (Cu) and uranium (U) element maps for the three complex samples** 3](#_Toc156226250)

[**Figure S2: Composite element map for the Cs-134 complex sample** 4](#_Toc156226251)

[**Figure S3: Adsorption rate plot of Cs onto different materials** 5](#_Toc156226252)

[**Figure S4: Adsorption rate plot of U onto ferrihydrite-coated quartz** 6](#_Toc156226253)

[**Table S1: Percentage difference for particles vs. artefacts** 7](#_Toc156226254)

[**Table S2: Average counts per second per mm^2^ for the three complex samples** 8](#_Toc156226255)

# **Figure S1: Copper (Cu) and uranium (U) element maps for the three complex samples**

**
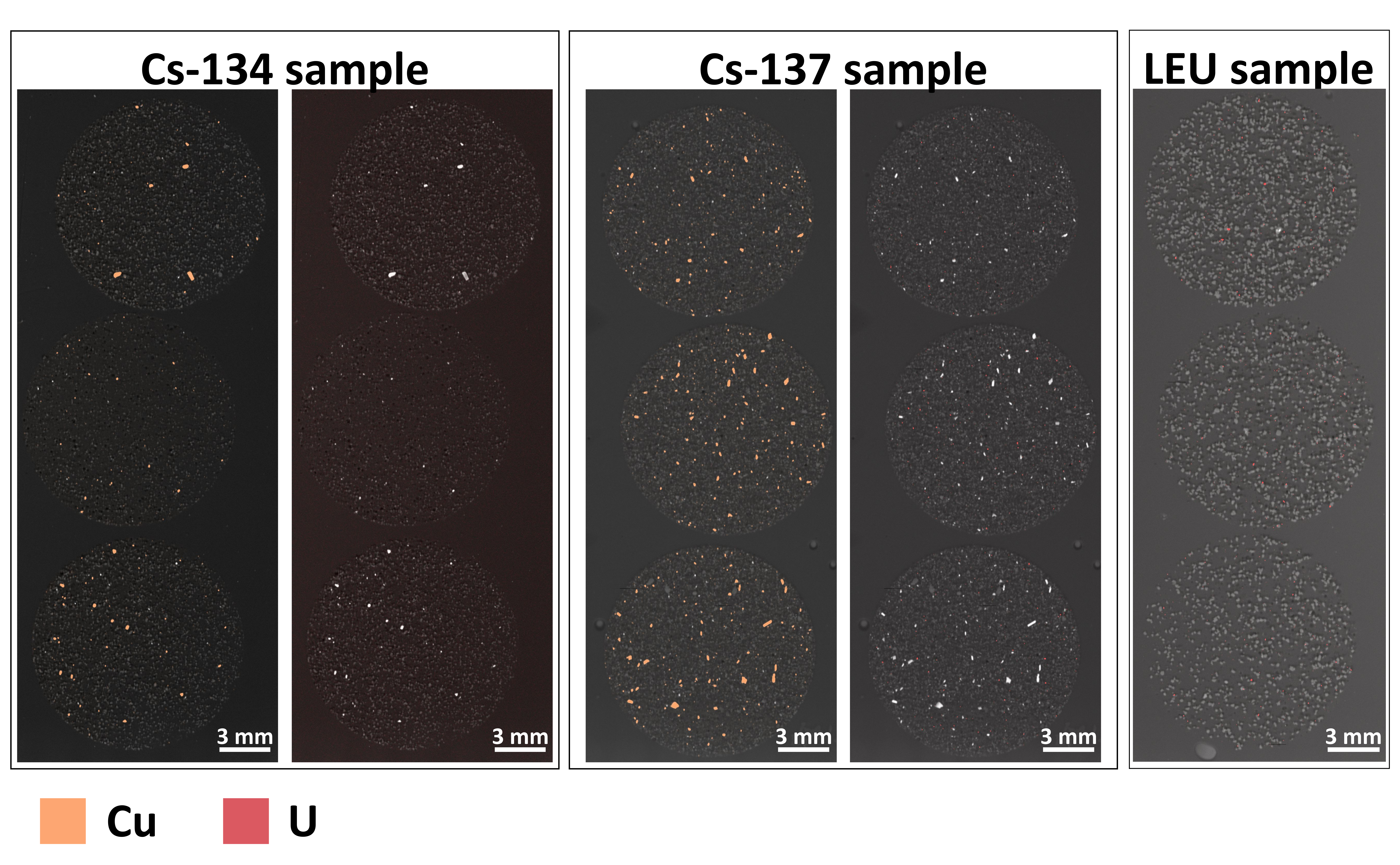
Figure S1.** Individual µXRF element maps for Cu (orange map on the left) and U (pink map on the right). Scale bars are found at the bottom of each map. For the LEU samples, only the U map is included as there were no Cu particles added to this sample.

# **Figure S2: Composite element map for the Cs-134 complex sample**


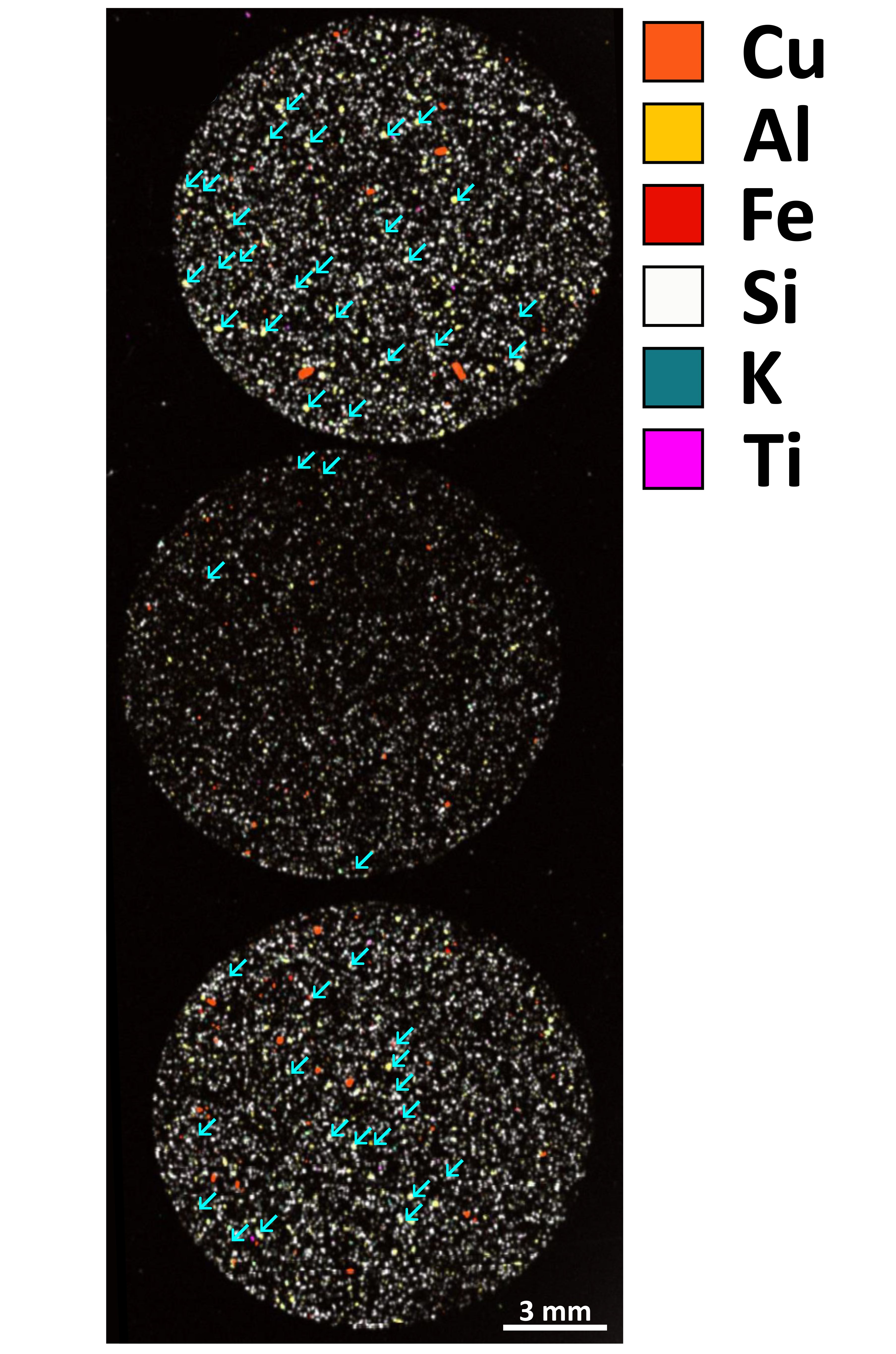


**Figure S2.** Composite element map of the Cs-134 sample. Cyan arrows indicate the regions in which the algorithm expresses false positive signals. Many of the false positive arrows point to grains containing aluminum (Al, yellow spots) or iron without copper (Fe, red spots). These elements belong to minerals such as illite and weathered biotite. Titanium is a contaminant found in the sample.

# **Figure S3: Adsorption rate plot of Cs onto different materials**


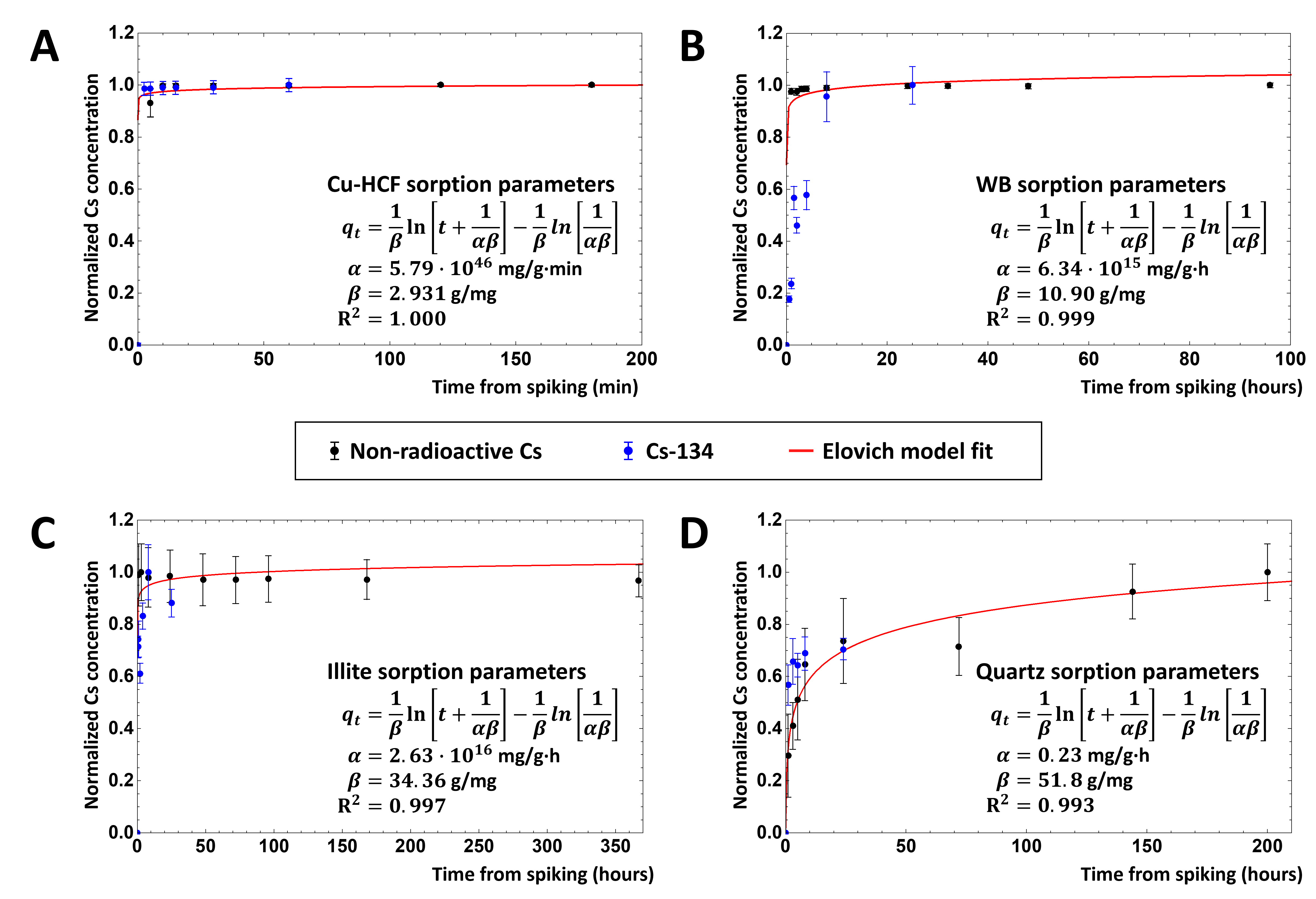


**Figure S3.** Adsorption rate plot of cesium (Cs) onto **(A)** copper hexacyanoferrate, **(B)** weathered biotite, **(C)** illite-smectite, and **(D)** quartz, respectively, at pH 7, room temperature, and pressure. The adsorption of non-radioactive Cs was carried out for longer time periods, as an initial pilot test for radiation safety. The data for the non-radioactive Cs (in black) were obtained by measuring aliquots of different time points via ICP-MS analysis. The error bars represent the calculated standard error from triplicates. Results from the non-radioactive Cs sorption were fitted to the Elovich model (red line). The radio-Cs adsorption was carried out for a shorter time (time to reach equilibrium in the pilot test). Cs-134 sorption data (in blue) were obtained by measuring the aliquots of different time points with gamma spectroscopy. The error bars were calculated from counting statistics. The Cs-134 data were not fitted to the Elovich model due to insufficient data points. Both plots were normalized against the maximum concentration for easier comparison.

# **Figure S4: Adsorption rate plot of U onto ferrihydrite-coated quartz**


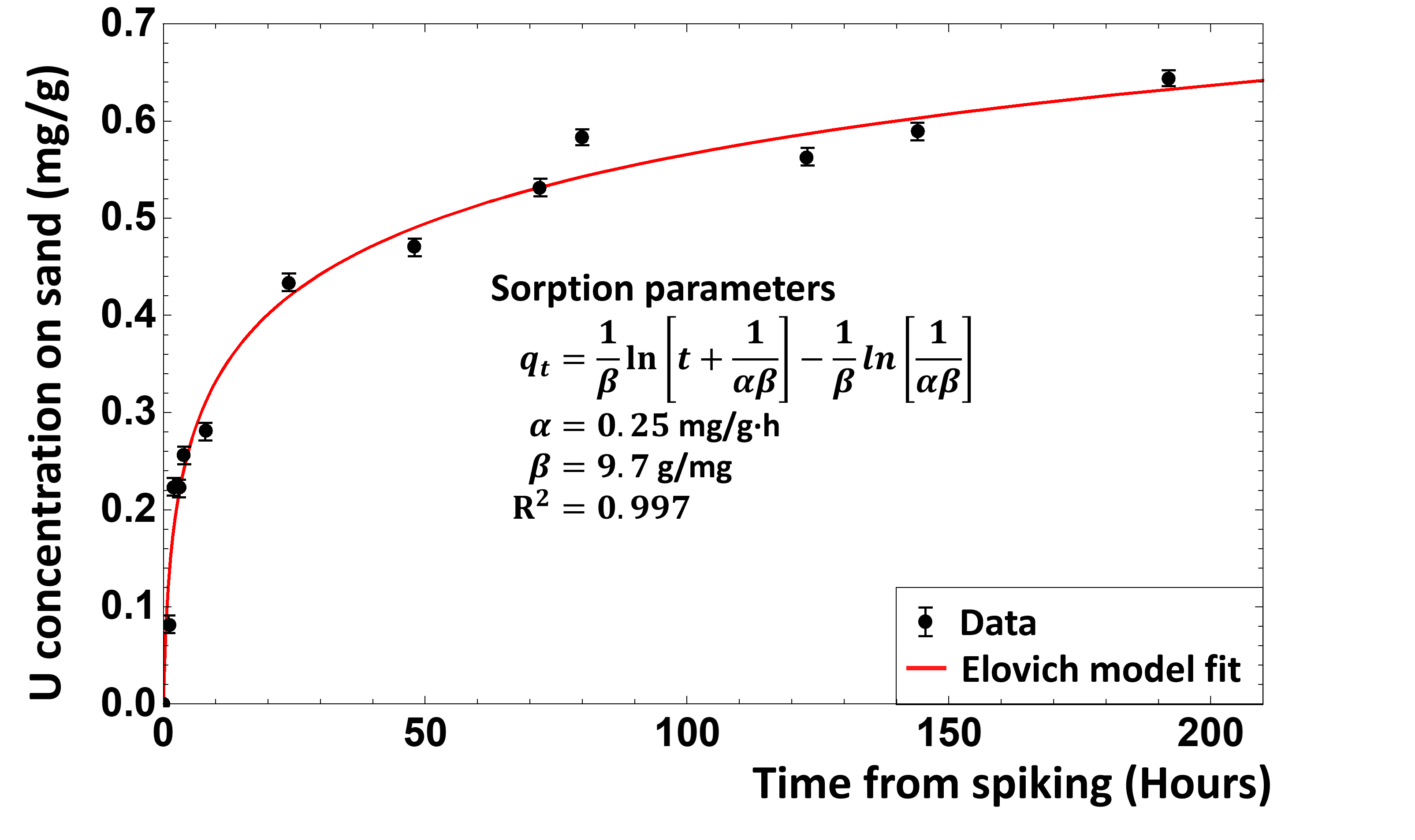


**Figure S4.** Adsorption rate plot of natural uranium (U) onto ferrihydrite-coated quartz, at pH 7.3, room temperature, and pressure. The data (in black) was obtained by measuring aliquots of different time points via ICP-MS analysis. The error bars represent the calculated standard error from triplicates. Results from the sorption were fitted to the Elovich model (red line).

# **Table S1: Percentage difference for particles vs. artefacts**

**Table S1.** Minimum and maximum counts from the time distribution plots in Figure 3, and the calculated percentage difference between both datapoints, respectively. All data were rounded to 3 significant figures.

| **Data** | **Minimum** | **Maximum** | **Percentage difference (%)** |
| --- | --- | --- | --- |
| Cs-134 particles | 0.775 | 0.909 | 15.9 |
| LEU particles | 0.656 | 0.789 | 18.4 |
| Dust/electronic artefacts | 0.360 | 0.841 | 80.1 |
| Air pocket artefacts | 0.400 | 0.900 | 76.9 |

# **Table S2: Average counts per second per mm^2^ for the three complex samples**

**Table S2.** Average counts per second per mm^2^ for a 5 × 5 mm^2^ region in the three complex samples, respectively.

| **Sample** | **Average counts (·10^−3^ counts/s/mm^2^)** |
| --- | --- |
| Cs-134 | 263 |
| Cs-137 | 4 |
| LEU | 0.5 |
